# Extended homozygous haplotypes at genes involved in brain development associated with autism

**DOI:** 10.1186/1753-6561-6-S6-P24

**Published:** 2012-10-01

**Authors:** Ping-I Lin

**Affiliations:** 1 Division of Biostatistics and Epidemiology, Department of Pediatrics, Cincinnati Children's Hospital Medical Center, Cincinnati, OH 45229, USA

## Background

Recent evidence has suggested that extended homozygous haplotypes (EHH) in several genomic regions may be associated with risk of psychiatric disorders (for example, schizophrenia). The phenomenon of EHHs may arise from recent positive selection, inbreeding, as well as recessive models.

## Materials and methods

We used Affymetrix 500K SNP arrays to search for EHH in 1,385 affected individuals and 1,498 unaffected individuals. The EHH was defined as at least 100 contiguous homozygous SNPs. To interrogate the associations between EHHs and autism, logistic regression analysis with generalized equation estimation model to adjust for intrafamily correlation was performed. We also examined if any of the associated EHHs were related to deletions by examining the data of copy number variants. Finally, we checked if any associated EHHs contained genes with signatures of recent positive selection in the Hapmap sample. We used the Sidak method to correct for multiple tests.

## Results

The best finding was obtained at the *HMLGCL1* gene (*P* = 3 × 10^–5^, odds ratio 0.15). The *HMLGCL1* gene has been found to be highly expressed in some brain regions. Other genes harbored in regions enriched with EHHs associated with autism include *ZFP91*, *CNTF*, *NAPL1* and *TLE4.* We also used the webtool Panther to assess if this set of genes is over-represented in any pathways. The results suggest that these genes collectively may be involved in the Wnt signaling pathway and neurodevelopment process. No remarkable evidence for recent positive selection was obtained for most of these loci, except the *TLE4* gene (*P* = 0.008).

## Conclusions

Taken together, these genes involved in brain development may harbor variants that exert a recessive effect on the risk of autism. Further work is warranted to replicate these findings in other populations.

